# Selection for small body size favours contrasting sex-specific life histories, boldness and feeding in medaka, *Oryzias latipes*

**DOI:** 10.1186/s12862-019-1460-x

**Published:** 2019-06-19

**Authors:** Beatriz Diaz Pauli, Sarah Garric, Charlotte Evangelista, L. Asbjørn Vøllestad, Eric Edeline

**Affiliations:** 1 Department of Biosciences, Centre for Ecological and Evolutionary Synthesis (CEES), University of Oslo, N-0316 Oslo, Norway; 2 Institut d’Ecologie et des Sciences de l’Environnement de Paris (iEES-Paris), Sorbonne Université, Université Paris Diderot, UPEC, CNRS, INRA, IRD, F-75252 Paris, France; 3 ESE Ecology and Ecosystem Health, INRA, Agrocampus Ouest, 35042 Rennes, France

**Keywords:** Age at maturation, Investment in reproduction, Growth, Boldness, Feeding behaviour, Resource availability, Size-selective mortality, Fisheries-induced evolution, Pace-of-life syndrome

## Abstract

**Background:**

Studying variation in life-history traits and correlated behaviours, such as boldness and foraging (i.e., pace-of-life syndrome), allows us to better understand how these traits evolve in a changing environment. In fish, it is particularly relevant studying the interplay of resource abundance and size-selection. These are two environmental stressors affecting fish in natural conditions, but also associated with human-induced environmental change. For instance, fishing, one of the most important threats for freshwater and marine populations, results in both higher mortality on large-sized fish and reduced population density.

**Results:**

Medaka, *Oryzias latipes,* from lines selected for large or small size over ten generations, were exposed individually to high or low food availability from birth to adulthood. Maturation schedules, reproductive investment, growth, boldness and feeding were assessed to evaluate the effect of size-selection on the pace of life, and whether it differed between food contexts (high and low). Different food abundance and size-selection resulted in diverse life histories associated with different feeding and boldness behaviour, thus showing different pace-of-life-syndromes. High availability of food favoured faster growth, earlier maturation and increased shyness. Selection for small size led to slower growth in both males and females. But, the life-history trajectory to reach such growth was sex- and food-specific. Under low food conditions, females selected for small size showed earlier maturation, which led to slower adult growth and subsequent low willingness to feed, compared to females selected for large size. No line differences were found in females at high food conditions. In contrast, males exposed to selection for small size grew slower both as juvenile and adult, and were bolder under both feeding regimes. Therefore, the response to size-selection was more sensitive to food availability in females than in males.

**Conclusions:**

We showed that size-selection (over ten generations) and resource abundance (over developmental time) led to changes in life history and behaviour. However, the effect of size-selection was sex- and context-specific, calling for precaution when drawing general conclusions on the population-level effects (or lack of them) of size-selective fishing. Conservation and management plans should consider this sex- and context-specificity.

**Electronic supplementary material:**

The online version of this article (10.1186/s12862-019-1460-x) contains supplementary material, which is available to authorized users.

## Background

Variation in life histories arises from different trajectories of survival, growth and fecundity that maximises fitness in different environments [1]. Somatic growth, time of maturation and reproductive investment are key factors in an individual’s life cycle and hence represent a life-history strategy [2–4]. Numerous environmental factors (e.g., resource abundance, temperature, inter- and intraspecific interactions) can affect growth and maturation [5, 6]. Studying how these factors affect variation in life-history strategies is particularly relevant to better understand the potential evolutionary adaptation of wild populations [7, 8].

In the particular case of fish, size-selective mortality and resource availability are two environmental stressors of interest. Selection on body size in fish can result from natural mortality, as predators normally target small individuals [9–11] or from fishing-induced mortality that is commonly higher on large individuals [12–14]. Food abundance is affected by many factors such as intraspecific competition, climate change and predation [15]. Increased predation (by predators or fishing) not only leads to selection on size, but further results in lower abundance in the population and thus increases food availability for the survivors [16]. This interplay between size-selection and resource availability on life-history traits is still poorly understood [17].

Size-selective mortality on large individuals, as that induced by fishing, leads to decreased life span. And hence, according to life-history theory, fast life histories are favoured – i.e., earlier maturation and increased reproductive investment [5, 14, 18, 19]. However, how size-selective fishing affects juvenile and adult growth remains unclear [5, 14]. Most empirical evidence shows no direct effect of size-selective fishing on growth [20], or an indirect effect resulting in reduced growth only after maturation [14]. Yet, such a response may not appear if resource availability is high [21]. Moreover, populations exposed to fishing would experience fast or slow growth, depending on the selectivity of the gear and the minimum size imposed [5, 22, 23].

Life-history diversity often entails co-variation with behaviour, as behaviour and physiology are the basis for the trade-off between current and future reproduction (referred as pace-of-life syndrome) [18, 24]. Fast life history is expected to be linked with behaviours that favour energy acquisition (e.g., foraging behaviour) and reproduction over survival (e.g., boldness), resulting in a fast pace of life [18, 24]. However, the few studies that have evaluated how behaviour was affected by size-selective mortality showed that fish exposed to positive size selection were less bold and less eager to forage [25, 26]. Behavioural and life-history co-variation are often sex- and context-dependent [24, 27]. Thus, knowledge on how the suit of life-history and behavioural traits changes due to size-selective mortality is still limited. Particularly, little is known about whether such changes are affected by the release from density-dependent food limitation following the reduction in abundance in harvested populations, and whether both sexes despite their different investment in reproduction are responding in a similar way.

Here we tested how two different size-selective mortality regimes (on large or small size) affected the pace-of-life syndrome in medaka (*Oryzias latipes*). In addition, we evaluated whether the result of size-selection differed under different food availability conditions. Wild medaka were used to produce two laboratory-reared lineages over ten generations with selection for either large or small standard body length at 75 days post-hatching (dph); here referred to as large-selected and small-selected lines, respectively [28]. The line selected for small size mimics the size-selective pressure induced by fishing, where large individuals are harvested and mainly the smaller individuals can reproduce. The large-selected line mimics natural size-selection, where predators feed on small individuals. Lines were selected under common-garden conditions with abundant food supply. Individuals from the 11th generation were reared in isolation under two different feeding regimes (high and low food availability). We fitted data on age and length to a biphasic (juvenile and adult) growth model that allowed studying trade-offs in energy allocation between growth, maturation and reproduction [29–31]. Females were measured every 2 weeks, while males every three, thus models were fitted separately for each sex. Once fish became mature, we assessed the feeding behaviour and boldness of each individual. Both behaviours have ecological validity, as they are respectively linked to foraging rate and risk of predation and hence related to fitness [32].

We expected individuals from the small-selected line to express 1) fast life histories (i.e., early maturation and high investment in reproduction), with growth only slowing down after maturation, and 2) behaviours associated with a fast pace of life, i.e., higher boldness and feeding rate compared to large-selected line [14, 18]. We also predicted that 3) individuals under low food availability would present slower pace of life relative to under high food [2, 33]. The effects of size-selection on behavioural and life-history traits would hold true at differing food abundances and for both sexes if food availability did not play a role in the selection process [17, 34], as expected under controlled equal and abundant food availability in the selection experiment. However, different reproductive investments between sexes may affect this expectation [27].

## Results

### Life-history traits

**Fig. 1:**
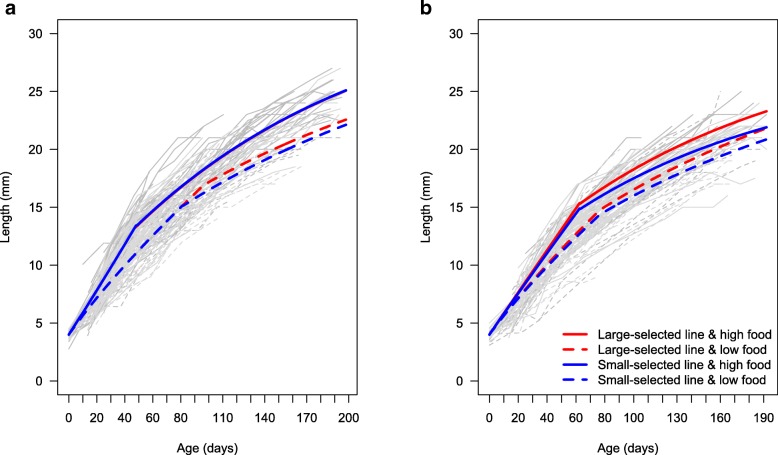
Growth trajectories from raw data (grey lines) and growth rates estimated by the growth models (coloured lines) for a) females and b) males. See parameters values in Tables 1 and 3

Both male and female medaka showed large inter-individual variation in growth and life-history parameters (age at maturation and maximal potential growth; Figs. 1a and b). Life-history traits differed between the two laboratory-reared lines (i.e., small-selected vs. large-selected) and the two food availability conditions applied during development (high vs. low; see Methods for details). Both low food availability and selection for small size led to smaller size, but the life-history trajectory leading there was context-specific (different between treatments) and different between sexes.

At the end of experiment (180 dph), fish in the high food availability treatment were larger compared to those fed low food in both sexes (Figs. 1a and b). In addition, fish from the large-selected line were larger at 180 dph compared to those originating from the small-selected line, although this was food-dependent in females (Fig. 1a). Overall, the effect of size-selection in females was absent in the juvenile phase of the growth curve (Fig. 1a). It was only observable in adult growth under low food conditions, when the earlier age at maturation of small-selected females resulted in a slower adult growth and hence smaller length at 180 dph (Fig. 1a; Table 1). Thus, the effect of line on female growth only occurred indirectly through its effect on maturation. The effect of size-selective mortality in males was evident, with juvenile and adult growth always being slower for small-selected males, and direct, as it was not affected through any other parameter.

**Table 1:** Female growth model structure and estimates (standard errors, SE) for *a*_mat_, *r*, and β

	Parameter estimates (SE)		
Treatment	*a*_mat_ (day)	*r*	β
Large-selected line & High Food	48 (1.7)	0.018 (0.001)	0.66 (0.007)
Small-selected line & High Food	48 (1.8)		
Large-selected line & Low Food	97 (4.5)	0.007 (0.0009)	0.53 (0.006)
Small-selected line & Low Food	81 (4.6)		

Age at maturation, *a*_mat_, reproductive investment, *r*, and growth allometric exponent, β. See Table 2 for treatment effect and statistics on parameter estimates

Average age at maturation (*a*_mat_) was 68 dph (SD = 24.5) in females and 71 dph (SD = 10.6) in males, which is within the range commonly observed for medaka (60–90 days) [35]. It was affected by the interaction of food and line in females, but not in males. As expected, females delayed maturation under low food conditions, but the delay differed between small- and large-selected females (Tables 1 and 2). At low food conditions, small-selected females matured earlier (81 dph) than large-selected females (97 dph), while at high food both lines matured at 48 dph (Tables 1 and 2). In contrast, maturation in males was not affected by size-selection, and only delayed under low food (Tables 3 and 4). Investment in reproduction was not affected by size-selection in either sex, as lower food led to a lower investment in reproduction in both sexes (Tables 2 and 4).

**Table 2:** Results from treatment effects on female growth model

	Coefficient	Std. Error	d.f.	*t*-value	*P*-value
*a*_mat_: (Large-sel. & H Food)	47.71	1.74	949	27.37	< 0.0001
*a*_mat_: Small-sel.	0.53	2.37	949	0.22	0.824
*a*_mat_: Low food	49.71	4.87	949	10.20	< 0.0001
*a*_mat_: Small-sel. & L Food	−16.87	6.57	949	−2.57	0.010
*r*	0.02	0.001	949	17.97	< 0.0001
*r*: Low Food	− 0.01	0.001	949	−7.79	< 0.0001
β: (Large-sel. & H Food)	0.66	0.007	949	100.56	< 0.0001
β: Low Food	−0.13	0.009	949	−15.24	< 0.0001

Output from non-linear mixed effect model for female biphasic growth with estimated coefficients, standard errors, *t* and *P* values, and degrees of freedom (d.f.). Small-selected line is referred as Small-sel., while Large-selected line is referred as Large-sel. High and low food are referred as H Food and L Food, respectively

**Table 3:** Male growth model structure and estimates (standard errors, SE) for *a*_mat_, *r*, and β

	Parameter estimates (SE)		
Treatment	*a*_mat_ (dph)	*r*	β
Large-selected line & High Food	63 (2.4)	0.016 (0.001)	0.63 (0.009)
Small-selected line & High Food			0.61 (0.008)
Large-selected line & Low Food	78 (3.9)	0.008 (0.001)	0.53 (0.011)
Small-selected line & Low Food			0.52 (0.011)

Age at maturation, *a*_mat_, reproductive investment, *r*, and growth allometric exponent, β. See Table 4 for treatment effects and statistics on parameter estimates

**Table 4:** Results from treatment effects on male growth model

	Coefficients	Std. Error	d.f.	*t*-value	*P*-value
*a*_mat_	62.70	2.38	622	26.32	< 0.0001
*a*_mat_: (H Food)	14.83	4.60	622	3.23	0.001
*r*	0.02	0.001	622.	14.88	< 0.0001
*r*: (H Food)	− 0.01	0.002	622	−5.20	< 0.0001
β: (Large-sel. & H Food)	0.63	0.009	622	65.94	< 0.0001
β: Small-sel.	−0.02	0.005	622	−2.88	0.004
β: Low food	−0.09	0.014	622	−6.96	< 0.0001

Output from non-linear mixed effect model for male biphasic growth with estimated coefficients, standard errors, *t* and *P* values, and degrees of freedom (d.f.). Small-selected line is referred as Small-sel., while Large-selected line is referred as Large-sel. High and low food are referred as H Food and L Food, respectively

The maximal potential growth rate (related to β; see Methods) describes the growth rate before the investment in reproduction is accounted for [29]. Higher values of β correspond to faster juvenile somatic growth rate, i.e., before any investment in reproduction takes place. Specifically, β is the exponent of the allometric relationship between growth rate and weight. It describes the allometric scaling of energy readily available to growth (i.e., net energy after expenditure on metabolism has been accounted for) [29]. Our average estimated value for β was 0.58 (SD = 0.06), which is similar to the 0.66 assumed in many growth models [29, 36]. For both males and females, β was lower – resulting in a slower juvenile growth – at low food conditions than at high food conditions, (Tables 1 and 3). In males there was also a direct effect of line on β, which was additive to the effect of food on β, but lower in magnitude. Large-selected males at high food conditions presented the highest value of β and hence the fastest juvenile growth rate, while small-selected males at low food had the lowest β and slowest juvenile growth rate (Table 3; Fig. 1b).

### Behavioural traits

Two different behaviours were assessed once the test fish reached maturity. Feeding behaviour was measured as the total number of bites the fish took at the supplied food, while boldness was related to time spent freezing at the bottom of the tank – the shorter freezing time the bolder an individual is [32]. Both behaviours were repeatable over time and across contexts (Additional file 1: Table S1). Feeding behaviour and boldness were evaluated as both total amount (bites on food or freezing time) and probability (of zero bites or zero freezing) due to the nature of the data (see Methods for details). Food availability and size-selection affected the behaviours and this was sex-specific (Table 5).

**Table 5:** Summary of the predictors affecting each behaviour, for males and females

Behavioural traits	Type of statistical test	Effects	
		Males	Females
Number of food bites	Zero-inflated negative Binomial	1	Food
	Zero-inflated Bernoulli	Food	Food + Line
Freezing time (Inverse of boldness)	Zero-inflated negative Binomial	Line	Food
	Zero-inflated Bernoulli	Line	1

Actual effects and *P*-values are given in the text. 1 means the parameter is unaffected by treatments

**Fig. 2:**
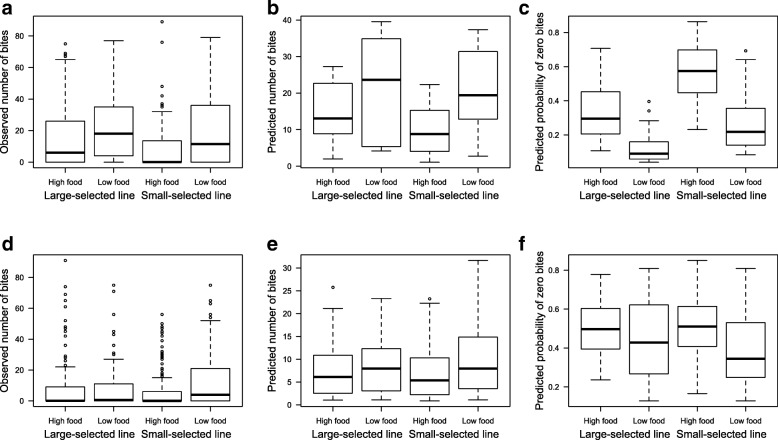
Number of bites taken by **a**–**c**) females and **d**–**f**) males under two food availabilities (High and Low food) and for both size-selection lines (Large- and Small-selected lines). In **a**) and **d**) the raw data are presented, i.e., the total number of bites observed, while **b**) and **e**) represent the model estimated total number of bites, and **c**) and **f**) show the model estimated probability of not biting at all during 5 min observations. Bar represent medians, lower and upper hinges of the box represent the first and third quartile, and whiskers show 1.5 times the interquartile range

Feeding rate was higher under low food conditions in both sexes (Fig. 2), but this effect was stronger in females than in males. Females reared at low food fed more than those at high food, as indicated by both their higher number of total food bites (Fig. 2b; low-high food comparison, estimate ± SE = 0.27 ± 0.10 bites, *z* = 2.72, *P* = 0.007) and lower probability to remain without eating (low-high food comparison: 3 times lower odds, *z* = − 4.48, *P* < 0.0001). Males at low food had only lower probability of no biting at all relative to high food males (low-high food comparison: 1.6 times less odds, *z* = − 2.81, *P* = 0.005). In addition, small-selected females presented reduced feeding activity (Fig. 2a–c) and they had higher probability of not eating at all compared to large-selected ones (small- large-selected comparison: 2.4 higher odds, *z* = 3.48, *P* < 0.001).

**Fig. 3:**
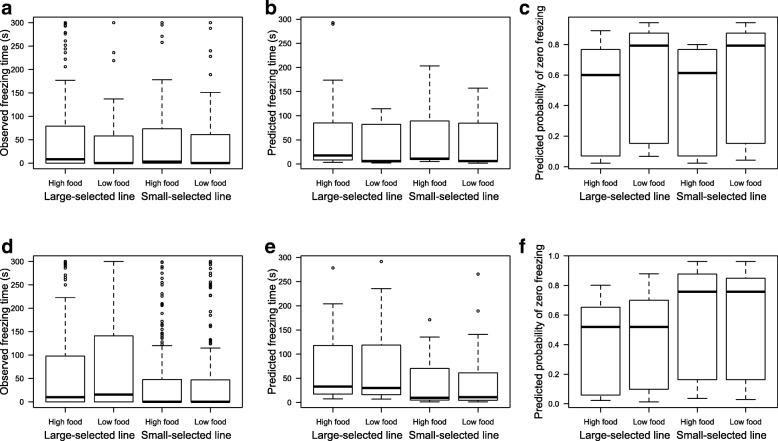
Freezing time – i.e., total amount of time fish remained immobile in the aquarium– in **a**–**c**) females and **d**–**f**) males, under two food availabilities (High and Low food) and for both size-selection lines (Large- and Small-selected lines). In **a**) and **d**) raw data are presented, i.e., the observed time in seconds, while **b**) and **e**) represent the model estimated freezing time, and **c**) and **f**) show the model estimated probability of not freezing at all during 5 min observations. Note that higher counts of freezing time in **b**) and **e**) are linked to lower probability of freezing 0 s in **c**) and **f**). Bar represent medians, lower and upper hinges of the box represent the first and third quartile, and whiskers show 1.5 times the interquartile range

Finally, total freezing time (used as a proxy for boldness) was affected by size-selection in males and by food availability in females (Fig. 3). Females at low food conditions were bolder (Fig. 3a–c) than females fed high food quantities, as the former had 1.6 times higher probability of not freezing at all (estimate ± SE = 0.48 ± 0.21 log(odds), *z* = 2.24, *P* = 0.025). Small-selected males were bolder – they froze 0.43 s less than males selected for large size (SE = 0.15, *z* = − 2.97, *P* = 0.003), hence they had higher probability of not freezing at all (Fig. 3 d–f; 1.8 higher odds, *z* = 3.50, *P* = 0.0005) relative to large-selected males.

## Discussion

Size-selection over ten generations affected life-history traits, boldness, and feeding behaviour. These traits were also affected by exposure to different food availabilities during development of each fish to a somewhat larger extent. Both the selection for small size (experienced by the parental generation) of fish and exposure to low food availability during development, led to shorter length at adulthood. However, the life-history traits (age at maturation, maximal potential growth and investment in reproduction) that led to such outcome were context-specific and differed between food availability and size-selection. Moreover, the effect of size-selection, but not the effect of food availability, was sex-specific.

It should be noted that comparisons between the two drivers evaluated here (i.e. size-selection and food availability) are challenging, as they were acting on different ecological processes (i.e. plasticity vs. evolution). Specifically, food availability affected the environment of the studied fish during their development from juvenile to adulthood, while size-selection was the stressor experienced by ten ancestral generations. Food availability and size-selection can lead also to evolutionary and plastic changes, respectively [33, 37], but here the experimental design limited those effects. Indeed, food availability was a proximate driver of plastic change in life-history traits, while size-selection could affect life history through genetic change (evolution), as it was performed under common garden conditions over ten generations [38–40]. Epigenetic inheritance cannot be ruled out, but it is likely not related to size-selection [40]. We warrant this simplification to better disentangle the effect of each treatment, before more complex interactions between treatments, through intraspecific competition should be assessed. In addition, sex differences could be due to differences in handling the sexes during the experiment due to time and space limitations. Males were measured every 3 weeks and water quality was maintained with partial water changes (for half of the males) and constant flow-through (on the other half), while females were measured every 2 weeks and water quality was maintained by constant flow-through of water. Even though these differences should not affect the effect of experimental treatments, but the statistical power, its effect cannot be completely ruled out.

### Effect of food

As expected, low food availability led to smaller size at adulthood and slower growth relative to high food availability in both sexes [2, 33, 41] . Low food availability led to higher willingness to feed and increased boldness (less freezing time) compared to high food availability, although the latter only happened in females. Thus, the hunger level might be the driver of increased boldness and increased willingness to eat, as seen elsewhere [42, 43]. Specifically, Magnuson [43] found that under limited food medaka became more aggressive to ensure access to food and maximal growth rate. Fast growth is generally associated with boldness and willingness to forage through evolutionary trade-offs [18], but these links often depend on environmental conditions [44]. In the present study, fast-growing fish at high food condition might have been feeding at a maximum ratio. Indeed, they showed lower immediate willingness or need to feed (no hunger and low appetite) during our observational assays, and lower boldness (at least in females). The links here between growth and behaviour are driven by hunger levels and are thus probably plastic.

### Effect of size-selection

Fish selected for small body size grew slower relative to those selected for large body size, as normally observed in other size-selective experiments on fish [25, 38, 39, 45, 46]. However, the overall shorter length at adulthood in small-selected fish, were achieved through different growth trajectories between sexes. Here, growth depended on the combination of the exponent of the maximal potential growth rate (related to β) and age at maturation.

Size-selection affected growth rates in males throughout their lives, but only during adulthood in females – this was due to size-selection effects on different parameters. Males experienced a direct effect of size-selection on growth, affecting only β – males selected for small size had lower values of β and thus grew slower. This, together with the lack of differences in maturation and reproductive investment between lines, led to different growth curves throughout the life of the fish. Females experienced a direct effect of size-selection on age at maturation under low food conditions. This led to an indirect effect of size-selection on growth, which resulted in equal juvenile growth between lines followed by a slowed down adult growth in small-selected females reared at low food. At high food conditions, females of both lines presented equal growth curves. Reduced age at maturation after selection for small size is expected from theory [14, 19] and observed in laboratory experiments [25, 34, 39, 45], but only occurred for females reared under low food conditions in our experiment.

The interaction between size-selection and food suggests that food availability played a role during the selection process in females [17, 34]. The higher investment in reproduction in females relative to in males may have resulted in females perceiving their food environment, during the selection process, as quantitatively low. This higher sensitivity to food in females also became apparent in our results on behaviour – the feeding and boldness of females were affected more by food than those of males. This is in concordance with earlier studies in medaka showing that females are more sensitive to fasting than males, resulting in reduced gonadosomatic index and fecundity [47]. Thus, it seems that females were only selected for small size under low food availability, and hence the response to selection is stronger under conditions similar to those experienced under the selection process [48]. Similar resource-dependent response to size-selection has been seen in other fish species [17, 49]. In the case of the killifish, *Rivulus hartii****,*** the females response to size-selection were also more sensitive to food availability than the males [17]. The context-specificity of life-history trajectories, which differed between sexes here, but also between species [25, 38, 39, 45, 46], indicate that the life history response to size-selection is more complex than often assumed.

Medaka selected for small size were bolder than medaka selected for large size. This was evident at least in males, which showed reduced juvenile and adult growth. Medaka females fed less when selected for small size, which presented early maturation and slow adult growth. Size-selection experiments with Atlantic silversides*, Menidia menidia*, have shown a reduced food consumption in fish selected for small size [26]. However, similar selection experiments have indicated reduced boldness in fish selected for small size [25, 26]. As noted earlier, the evolutionary link between fast growth and boldness is common mainly when predation is high and resources are limited [44]. For instance, in medaka exposed to low food, higher aggression (commonly correlated with boldness [32]) was linked with higher growth rate, but this link disappeared when food supply was high [43]. When resources are abundant, low activity and boldness leads to higher growth as predicted by the allocation model [50]. Overall, we observed that changes in life-history parameters due to size-selection also led to changes in behaviour, which were consistent over time and among context [51].

Medaka do not present drastic morphological sexual dimorphism [35], but males and females present behavioural and physiological differences. Both sexes present courtship and competitive behaviours, but these are more evident in males [43, 52], while females invest more in reproductive tissues [53]. Moreover, external factors (e.g., food, temperature, pollutants) seem to alter reproductive investment in females (but not behaviour) and aggressiveness in males (but not reproduction or to a lower extent) [47, 54–57]. Overall, this sexual dimorphism in sensitivity to external stressors could explain the sexual differences observed in the present study. Moreover, our results highlight the need of assessing sexual differences while evaluating life-history and behavioural traits.

Here we show that the effect of size-selection, such as the one induced by fishing, on life history can entail behavioural changes. Boldness and foraging are at the core of predator-prey interactions, as they determine the effects of consumers on their prey and are affected by the presence of predators [58]. Therefore, changes in behaviour due to human-induced size-selection can in turn affect the resource community and ecosystem processes through different pathways, such as alteration of the strength of the trophic cascade [59]. Moreover, the links between life history and behaviour are more complex than often assumed and dependent on sex and environmental conditions. A better understanding on how size-selective mortality affects this suit of traits is not only relevant for management and conservation of exploited species, but allow us to predict further ecosystem consequences. We suggest that future mesocosm-based experiments should assess whether differences in these size-dependent correlated traits can translate to changes in the trophic cascade to better evaluate the ecosystem impacts of size-dependent mortality. Moreover, such experiments could allow the interplay of food availability and size-selection and hence assess the context-dependency of such ecological variations.

## Conclusions

In the present study, both sexes displayed smaller size and slower growth when exposed to selection for small size, similar to the size-selection induced by fishing. However, life-history strategy and pace-of-life syndromes were sex- and context-specific in the present study.

Small-selected females showed a fast pace of life under low food conditions, i.e., early maturation and fast juvenile growth – only slowed down after maturation. Slower adult growth was linked to reduced feeding rate. However, small-selected males grew slower throughout life linked with a higher boldness relative to fish selected for large size. Sex differences may be due to differences in investment in reproduction and food requirements during the selection process.

Conservation plans concerned with size-selectivity (e.g., fishing or introduction of novel predators) should consider that a suit of behavioural and life-history traits, rather than only size, are responding to the new selection. In addition, the interplay between size-selection and resource availability should be evaluated to better account for the impact on ecosystem functioning and services.

## Methods

### Selection lines

Individuals used in this study were the offspring of the 10th generation (F10) produced by a size-selection experiment performed in the laboratory (see [28] for details on experimental protocol). Briefly, the selection for small standard length (SL ± 1 mm) (referred as Small-selected line) mimicked the selection imposed by fishing, where large individuals are removed and only small individuals are allowed to breed. Selection for large individuals (i.e., large SL, referred as Large-selected line) represents natural mortality in the wild. Specifically, at 60 days post hatching (dph), the ten brother-sister families with the shortest or largest average length were selected for the small-selected and large-selected lines, respectively. At 75 dph, individual size-selection took place and the breeders (two males and two females per family) for the next generation were chosen. The largest and the smallest individuals per family were chosen for the line selected for large size and selected for small size, respectively.

### Fish rearing and feeding experiment

At generation F10, we randomly chose five families from each line to produce at least 64 fish per line and per feeding treatment (*N* = 256). F10 breeders were 90 dph when eggs were collected and kept in an incubator until the larvae hatched. The incubator was checked for daily hatchlings and hatching day was recorded for all larvae. Larvae hatched on the same day were kept together within family in 3 L tanks for 2 weeks. At 14 dph, each surviving individual larva was randomly assigned to one of two feeding regimes and housed in isolation in 1 L tanks.

Every 2 weeks females were measured for SL with a measuring board and weighted (W ± 0.001 g), while males were only measured and weighted every 3 weeks due to time limitation. The first two measurements (at 14 and 28 dph) were obtained with ImageJ (version 1.51 s; [60]) from photographs of the larvae placed in a petri dish filled with water (no weight was taken), as the larvae were too small to be handled otherwise. Later, individuals were anaesthetised (Metacaine; Sigma; following [35] protocols) to minimise stress during handling. At the end of the experiment 259 fish were included in the growth analysis as they had at least four measurements of size. Total number was not balanced among sexes and treatments. Specifically, we obtained growth trajectories from a total of 143 females (39 from large-selected line in high food, 36 from small-selected line in high food, 28 from large-selected line in low food and 40 from small-selected line in low food) and 116 males (19 from large-selected line in high food, 41 from small-selected line in high food, 20 from large-selected line in low food, and 36 from small-selected line in low food).

Fish were fed once a day (morning) with quantified amounts of newly hatched *Artemia salina* based on previous experiments [61, 62] During the first 2 weeks, from birth until the initiation of the treatments, all fish received the same food quantities (0–14 dph: 0.01 ml of filtered, undiluted *Artemia* per fish). Then, high and low food levels were applied and increased every 2 weeks. These quantities of food delivery were chosen to sustain two different growth rates, with the high level being double the low level [61, 62]. Specifically, high food level consisted of 0.01 mL of *Artemia* at 15–28 dph and reached 0.05 mL from 143 dph until the end of the experiment.

In the 1-L tanks, females were housed in a flow-through system, while only half of the tanks holding males where housed in the same flow-through system. Due to space limitations, the other half of male tanks was housed outside of the flow-through system (partial water changes every 2 weeks), but still inside the same lab (same temperature of 26 degrees Celsius). These males were only outside of the flow-through system during the growth part of the experiment, and not during the behavioural assessment. In addition, to minimise this effect, males were randomly rotated every 3 weeks between inside and outside the flow-through system. Finally, measurements were more frequent in females than males and, because they were also exposed to constant flow-through, data sets are analysed separately. At the end of the study all individuals were anaesthetised with Metacaine (Sigma; following [35] protocols) and later euthanised with an overdose of Metacaine as they were included in a study on pituitary gene expression.

### Estimation of life history traits

The life-history parameters, age at maturation, investment in reproduction and growth rate were estimated using the Quince-Boukal biphasic growth model [29, 30]. This growth model fits better juvenile and adult growth curves compared to other commonly used growth models for fish, and has proven useful for generating management advice [36]. The model follows a continuous function with a smooth transition between the juvenile and adult growth phase. This transition is due to allocation of energy to reproduction. It assumes that the maximal potential growth rate scales allometrically with body size.

The formulation assumes that juveniles allocate all surplus energy into growth (i.e., the investment in reproduction *r*_a_ = 0). Juvenile growth curve for length, at age, *a*, *L*_a_, follows:

1 $$ {L}_a=\sqrt[\left(1-\beta \right)\alpha ]{L_0^{\left(1-\beta \right)\alpha }+c\left(1-\beta \right){b}^{-\left(1-\beta \right)}}a $$

The adult growth rate considers the investment in reproduction, *r*, of the mature individuals, whose age is larger than their age at maturation (*a* > *a*_mat_) and the weight-age growth curve follows:

2 $$ {L}_a=\sqrt[\left(1-\beta \right)\alpha ]{R^{a-{a}_{mat}}\left({L}_0^{\left(1-\beta \right)\alpha }+H{a}_{mat}\right)+\frac{RH}{1-R}\left(1-{R}^{a-{a}_{mat}}\right)} $$

where *H* = *c*(1 − β) *b*^−(1 − β)^, *R* = 1/[1 + (1 − β) *r*], assuming the conversion factor between somatic and gonadic investment, *q*, in [29] to be 1 as in [36, 63]. *L*_0_ is length at birth, *c* and β are the intercept and exponent in the allometric relationship of growth rate with weight, d*W*/d*t* = *c W*^*β*^ – which is also referred as maximal potential growth rate. b and α are the intercept and exponent of the allometric relationship between weight and length, *W* = *b L*^α^. See [29] for all details in formulation.

The coefficient, *b*, and exponent, α, of the allometric relationship of weight, *W*, with length, *L*, were estimated with the data prior running the growth model. The values obtained from a regression model with log-weight and log-length were α = 2.7 and *b* = 0.04 mg mm^-2.7^, which were used for both males and females. Length at birth, *L*_0_, was obtained from a subsample of the individuals (25 females and 22 males) that were photographed and measured at 0 dph. Length at birth did not differ between sexes (F_44, 1_ = 0.67, *P* = 0.5) or lines (F_44, 1_ = 0.36, *P* = 0.7). The mean length at 0 dph was 3.9 ± 0.4 mm, thus *L*_0_ = 4.0 mm was used in the model for both males and females. The linear models were performed with the “stats” R package [64].

Growth curves were estimated separately for males and females, as sexes normally differ in their life-history optima and considering together might impede the study of pace-of life syndrome [27], but also due to the differences in experimental handling between sexes. Thus, here we aimed at studying the effect of size-selection on growth in both sexes without directly comparing sexes. For each sex, the growth model estimated age at maturation *a*_mat_, reproductive investment *r*, and the exponent in the allometric growth rate-weight relationship β. To improve model convergence the coefficient in the allometric growth rate-weight relationship was fixed to *c* = 0.15 mg^1- β^ day^− 1^. Initial exploration of the data showed that this value was the most appropriate for our data and changes in this value with an increase or decrease of 10% did not qualitatively change the results. This scaling coefficient is species-specific [65], it has been estimated for another small freshwater fish, guppy *Poecilia reticulata* (*c* = 9–14 mg^1- β^ day^− 1^; [45]).

All statistical analyses were performed with the R software (version 3.5.0; [64]). The parameters were estimated from a non-linear mixed effect model in the R package “nlme” (version 3.1.137; [66]) with fish identity as random effect on *r* and β for males and on β only for females. The random structure was chosen following recommendations from [67]. Models included a residual autocorrelation structure ARMA (0,2), chosen according to guidelines in [67]. Line, food treatment and their interaction were tested as fixed effects on *a*_mat_, *r*, and β for both sexes separately. All possible models were run and model selection was done by comparing them with AIC (Akaike information criterion). The model yielding the lowest AIC was considered the best-ranked model in the Kullback–Leibler information [68]. However, for males this model was further simplified through hypothesis testing and non-significant predictors were dropped one by one to obtain a more parsimonious model [67].

### Behavioural traits

Behavioural observations took place on mature fish (mean age = 124 dph ± 33 SD for females and 131 dph ± 32 sd for males) in three different settings: 1) Control conditions where fish were fed *Artemia* undisturbed, 2) Novel conditions where fish were fed a novel food source (4 pellets; JBL NovoGrano Mix) undisturbed, and 3) Threatening conditions where fish were fed *Artemia* immediately after being netted out of the water for 2 s as threat stimuli. Each fish was exposed to the three conditions inside their tank in a randomized order, each exposition replicated twice. Only one condition was tested per day and the second replicate took place 1 week after the first one. The six measurements (three conditions repeated twice) of each behaviour (i.e. feeding and boldness; see details below) ensured that we considered consistent intrinsic individual variation in behaviour [51]. However, the effect of time and experimental condition was not the main focus of the study, thus details on the experimental set up and across-context repeatability can be found in the supplementary material (Additional file 1: Tables S1–S3).

During 5 min of observations we counted 1) number of bites to the supplied food (*Artemia* or pellets) as a measurement of willingness to feed, and thus is associated with foraging on prey [26], and 2) total time frozen at the bottom of the tank, as a proxy of boldness, which is related to predation risk and survival in natural conditions [32]. A fish that spends more time immobile is considered less bold than one that spends little time immobile [32]. Both behaviours are ecologically relevant, as they are linked to interactions with prey and predators [58]. Behaviour tests were performed on 80 females and 107 males, as only these were available at the time.

The two behaviours were analysed with generalized mixed effect models using the R package “glmmTMB” [69] with measurement nested within fish identity and experimental condition included as two separate random effect on model intercept. Full models contained size-selection (Large-selected vs. Small-selected), food (High food vs. Low food) and their interaction as fixed effects. The analyses of number of bites and freezing time (measured as an integer count of seconds) were performed following negative binomial distribution and allowing for zero inflation, as 33 and 49% of the data for number of bites were zeros for females and males respectively, and 52% of the values for freezing time were zeros for both males and females. Negative binomial distribution was used due to overdispersion observed with Poisson distribution. The final model was the one ranked with the lowest AIC. The residuals from all the final models were evaluated following [67] and fulfilled all the requirements. In addition, simulations showed that the zero-inflated models represented well the data; particularly the models estimated an equivalent number of zeros as in the original data on average in 40% of the simulations [70]. It should be noted that the analyses with negative binomial distribution and zero inflation evaluate each behaviour in two ways: 1) total amount of bites or seconds frozen, and 2) probability of zero bites or zero seconds frozen. Thus, the effects of size-selection and food are evaluated for both cases.

## Additional file

Additional file 1: Detailed experimental set up for the behavioural assessment. Behavioural repeatability (Table S1) and effect of experimental conditions on male (Table S2) and female (Table S3) behaviours. (DOCX 32 kb)

## Data Availability

The datasets supporting the conclusions of this article are available in the figshare repository: doi: 10.6084/m9.figshare.8255873.v2 [71].

## Abbreviations

AIC: Akaike information criterion
*a*_mat_: age at maturation
*b*: Intercept of the allometric relationship between weight and length
*c*: Intercept of the allometric relationship between growth rate and weight
dph: Days post-hatching
F10: 10th generation
L: Length
L_0_: Length at birth
*r*: reproductive investment
SL: Standard length
W: Weight
α: Exponent of the allometric relationship between weight and length
β: Exponent of the allometric relationship between growth rate and weight
