# Supplementary material for: Selection for small body size favours contrasting sex-specific life histories, boldness and feeding in medaka, Oryzias latipes
Source: BMC Evol Biol. 2019 Jun 19;19:127. doi: 10.1186/s12862-019-1460-x (PMC6585084; doi:10.1186/s12862-019-1460-x)
Supplement: Supplementary file 1 — Detailed experimental set up for the behavioural assessment. Behavioural repeatability (Table S1) and effect of experimental conditions on male (Table S2) and female (Table S3) behaviours. (DOCX 32 kb) [file 12862_2019_1460_MOESM1_ESM.docx]

**Supplementary material**

Selection for small body size favours contrasting sex-specific life histories, boldness and feeding in medaka, *Oryzias latipes*

.

Beatriz Diaz Pauli^1*^, Sarah Garric^2^, Charlotte Evangelista^1^, L. Asbjørn Vøllestad^1^ and Eric Edeline^2,3^

**Detailed experiment set up for the behavioural assessment**

Behavioural observations took place on mature fish (mean age = 124 dph ± 33 sd for females and 131 dph ± 32 sd for males) in three different settings: in 1) Control conditions where fish were fed *Artemia* undisturbed, 2) Novel conditions where fish were fed a novel food source (4 pellets; JBL NovoGrano Mix) undisturbed, and 3) Threaten conditions where fish were fed *Artemia* immediately after being netted out of the water for 2 seconds as threat stimuli. Each fish was exposed to the three conditions inside their aquaria twice in a randomized order. Only one condition was tested per day and the second round took place one week after the first one. The six measurements of each behaviour (three conditions repeated twice) ensured that we considered consistent intrinsic individual variation in behaviour as advised in [1].

A 0.5% solution of unfiltered *Artemia* in water was prepared at the beginning of the behavioural assay. This solution was inspected under the stereomicroscope and modified accordingly until we counted approximately 20 brine shrimps per drop of solution. For each day’s solution we inspected three drops obtaining on average 23.6 ± 5.1 *Artemia* per drop. The behavioural observations lasted for five minutes since the fish received the food (1 drop of *Artemia* solution or 4 pellets). During the 5 minutes of observation it was counted 1) number of bites to the supplied food, 2) total amount of time eating detritus from the bottom of the tanks, rather than eating the supplied food (i.e., *Artemia*, in Control and Threaten conditions, or pellets in Novel conditions), and 3) total time frozen at the bottom of the tank. The amount of time frozen, i.e., time immobile after a threat in the aquarium, is a proxy for boldness. A fish that spends more time immobile is considered less bold than one that spends little time immobile [2]. Behaviour tests were performed on 80 females and 107 males, as only these were available.

The repeatability of all the behaviours were calculated following a Poisson distribution corrected for overdispersion using the R package “rptR” [3]. This package estimates repeatability based on intra-class correlations. Repeatabilities were adjusted for the effect of the different experimental conditions. Confidence intervals were estimated from 1000 parametric bootstraps and statistical significance against H0: *R* = 0 is tested by likelihood ratio [3]. See table S1 for results.

All behaviours were affected by the experimental conditions. Overall, fish in control conditions fed more than in the other conditions, but they ate more the known food source after a threat than a novel food source in undisturbed conditions. In addition, as expected fish spent more time frozen after a threat than when they were undisturbed (Table S2 and S3). The effect of experimental condition was controlled in the final results by including this variable as random factor in the models.

| Table S1. Repeatability, *R*_adj_ (adjusted for experimental conditions), 95% bootstrapped confident intervals, CI, Likelihood ratio test statistic, *D* and significance of individual effects, *P* value, for each measured behavioural trait in males and females. | | | | | | | | | |  |
| --- | --- | --- | --- | --- | --- | --- | --- | --- | --- | --- |
|  | Females | | | | Males | | | |  |  |
| Behavioural trait | *R*_adj_ | CI | *D* | *P* value | *R*_adj_ | CI | *D* | *P* value | |  |
| Number of food bites | 0.26 | 0.13 – 0.39 | 25.2 | < 0.0001 | 0.25 | 0.12 – 0.34 | 43.8 | < 0.0001 | |  |
| Freezing time | 0.08 | 0 –0.16 | 2.97 | 0.04 | 0.17 | 1.8*10^-10^ – 0.2 | 18 | < 0.0001 | |  |

| Table S2. Models for each male behavioural trait with estimated coefficients, standard errors, *z* and *P* values. In models based on logistic regression the coefficients are expressed effects in log (odds) and the odds ratios are also given. In models based on count data the estimates are given in seconds. To facilitate interpretation reproductive investment, *r*, have been standardized to zero mean and unity standard deviation. Mean and SD for *r* is 0.015 ± 0.004. | | | | | | |
| --- | --- | --- | --- | --- | --- | --- |
| Number of food bites | *Zero-inflated Bernoulli distribution* |  | Estimate (s) | Std. Error | *z* value | *P* value |
|  | (Intercept) |  | 3.09 | 0.10 | 31.08 | < 2*10^-16^ |
|  | Exp Cond: Novel |  | -1.8 | 0.15 | -12.23 | < 2*10^-16^ |
|  | Exp Cond: Threat |  | -0.32 | 0.14 | -2.31 | 0.02 |
|  | *Zero-inflated negative binomial distribution* | Odds ratio | Estimate (log(odds)) | Std. Error | *z* value | *P* value |
|  | (Intercept) |  | -0.31 | 0.19 | -1.64 | 0.10 |
|  | Food: Low | 0.58 | -0.55 | 0.20 | -2.72 | 0.007 |
|  | Exp Cond: Novel | 1.45 | 0.37 | 0.27 | 1.4 | 0.16 |
|  | Exp Cond: Threat | 2.52 | 0.93 | 0.23 | 3.92 | 9*10^-5^ |
|  | *r* | 1.31 | 0.27 | 0.1 | 2.69 | 0.007 |
| Freezing time | *Zero-inflated Bernoulli distribution* |  | Estimate (s) | Std. Error | *z* value | *P* value |
|  | (Intercept) |  | 4.25 | 0.16 | 26.09 | < 2*10^-16^ |
|  | Line: Low |  | -0.43 | 0.14 | -3.00 | 0.002 |
|  | Exp Cond: Novel |  | -0.28 | 0.18 | -1.58 | 0.11 |
|  | Exp Cond: Threat |  | 0.84 | 0.15 | 5.68 | <1*10^-8^ |
|  | *Zero-inflated negative binomial distribution* | Odds ratio | Estimate (log(odds)) | Std. Error | *z* value | *P* value |
|  | (Intercept) |  | 0.40 | 0.20 | 1.99 | 0.05 |
|  | Line: Low | 2.52 | 0.93 | 0.21 | 4.34 | 1*10^-5^ |
|  | Exp Cond: Novel | 1.27 | 0.24 | 0.23 | 1.04 | 0.30 |
|  | Exp Cond: Threat | 0.04 | -3.11 | 0.27 | -11.37 | < 2*10^-16^ |

| Table S3. Models for each female behavioural trait with estimated coefficients, standard errors, *z* and *P* values. In models based on logistic regression the coefficients are expressed effects in log (odds) and the odds ratios are also given. In models based on count data the estimates are given in seconds. | | | | | | |
| --- | --- | --- | --- | --- | --- | --- |
| Number of food bites | *Zero-inflated Bernoulli distribution* |  | Estimate (s) | Std. Error | *z* value | *P* value |
|  | (Intercept) |  | 3.37 | 0.08 | 39.84 | < 2*10^-16^ |
|  | Food: Low |  | 0.27 | 0.1 | 2.75 | 0.006 |
|  | Exp Cond: Novel |  | -2.01 | 0.14 | -14.75 | < 2*10^-16^ |
|  | Exp Cond: Threat |  | -0.29 | 0.10 | -2.78 | 0.005 |
|  | *Zero-inflated negative binomial distribution* | Odds ratio | Estimate (log(odds)) | Std. Error | *z* value | *P* value |
|  | (Intercept) |  | -1.45 | 0.27 | -5.40 | 6*10^-8^ |
|  | Line: Low | 2.57 | 0.94 | 0.26 | 3.57 | 0.0003 |
|  | Food: Low | 0.29 | -1.25 | 0.27 | -4.56 | 5*10^-6^ |
|  | Exp Cond: Novel | 1.63 | 0.49 | 0.39 | 1.26 | 0.21 |
|  | Exp Cond: Threat | 4.05 | 1.4 | 0.29 | 4.85 | 1*10^-6^ |
| Freezing time | *Zero-inflated Bernoulli distribution* |  | Estimate (s) | Std. Error | *z* value | *P* value |
|  | (Intercept) |  | 3.63 | 0.21 | 17.57 | < 2*10^-16^ |
|  | Exp Cond: Novel |  | -0.26 | 0.29 | -0.9 | 0.37 |
|  | Exp Cond: Threat |  | 1.14 | 0.21 | 5.31 | <1*10^-04^ |
|  | *Zero-inflated negative binomial distribution* | Odds ratio | Estimate (log(odds)) | Std. Error | *z* value | *P* value |
|  | (Intercept) |  | 1.03 | 0.23 | 4.4 | 0.00001 |
|  | Exp Cond: Novel | 0.84 | -0.18 | 0.35 | -0.53 | 0.6 |
|  | Exp Cond: Threat | 0.03 | -3.68 | 0.37 | -9.97 | < 2*10^-16^ |
|  | Food: Low | 2.25 | 0.81 | 0.3 | 2.67 | 0.008 |

**References**

1. Killen SS, Adriaenssens B, Marras S, Claireaux G, Cooke SJ. Context dependency of trait repeatability and its relevance for management and conservation of fish populations. Conserv Physiol. 2016;4:cow007.

2. Réale D, Reader SM, Sol D, McDougall PT, Dingemanse NJ. Integrating animal temperament within ecology and evolution. Biol Rev. 2007;82:291–318.

3. Stoffel MA, Nakagawa S, Schielzeth H. rptR: repeatability estimation and variance decomposition by generalized linear mixed-effects models. Goslee S, editor. Methods in Ecology and Evolution. 2017;8:1639–44.
